# Racial Disparities-Associated COVID-19 Mortality among Minority Populations in the US

**DOI:** 10.3390/jcm9082442

**Published:** 2020-07-30

**Authors:** Donald J. Alcendor

**Affiliations:** Center for AIDS Health Disparities Research, Department of Microbiology, Immunology, and Physiology, School of Medicine, Meharry Medical College, Nashville, TN 37208, USA; dalcendor@mmc.edu

**Keywords:** coronavirus, COVID-19, infection, mortality, minorities, health disparities, health inequities, African Americans, Hispanics/Latinos, non-Hispanic Whites

## Abstract

Severe acute respiratory syndrome coronavirus 2 (SARS-CoV-2), a betacoronavirus that causes the novel coronavirus disease 2019 (COVID-19), is highly transmissible and pathogenic for humans and may cause life-threatening disease and mortality, especially in individuals with underlying comorbidities. First identified in an outbreak in Wuhan, China, COVID-19 is affecting more than 185 countries and territories around the world, with more than 15,754,651 confirmed cases and more than 640,029 deaths. Since December 2019, SARS-CoV-2 transmission has become a global threat, which includes confirmed cases in all 50 states within the United States (US). As of 25 July 2020, the Johns Hopkins Whiting School of Engineering Center for Systems Science and Engineering reports more than 4,112,651 cases and 145,546 deaths. To date, health disparities are associated with COVID-19 mortality among underserved populations. Here, the author explores potential underlying reasons for reported disproportionate, increased risks of mortality among African Americans and Hispanics/Latinos with COVID-19 compared with non-Hispanic Whites. The author examines the underlying clinical implications that may predispose minority populations and the adverse clinical outcomes that may contribute to increased risk of mortality. Government and community-based strategies to safeguard minority populations at risk for increased morbidity and mortality are essential. Underserved populations living in poverty with limited access to social services across the US are more likely to have underlying medical conditions and are among the most vulnerable. Societal and cultural barriers for ethnic minorities to achieve health equity are systemic issues that may be addressed only through shifts in governmental policies, producing long-overdue, substantive changes to end health care inequities.

## 1. Introduction

Coronavirus disease 2019 (COVID-19) is caused by the severe acute respiratory syndrome coronavirus 2 (SARS-CoV-2) [1,2,3,4]. To date, seven human coronaviruses (HCoVs) have been identified, including two α-CoVs (HCoV-229E and HCoV-NL63) and five Betacoronaviruses (β-CoVs) (HCoV-OC43, HCoV-HKU1, severe acute respiratory syndrome CoV [SARS-CoV], Middle East respiratory syndrome CoV [MERS-CoV], and most recently β-CoV SARS-CoV-2 [COVID-19]) [5,6,7,8,9]. The World Health Organization (WHO) has classified COVID-19 as a β-CoV of group 2B [10]. CoVs cause respiratory, enteric, hepatic, and neurological diseases in various animal species, including camels, cattle, cats, and bats [11,12]. The β-CoV lineages HCoV-OC43 and HCoV-HKU1 typically are associated with self-limiting upper respiratory infections in immunocompetent hosts and occasionally lower respiratory tract infections in immunocompromised hosts and the elderly [13,14]. Examination of the viral evolution reveals that bats and rodents are gene sources for most α-Covs and β-CoVs, whereas avian species are the proposed gene sources of most δ-CoVs and γ-CoVs [15]. CoVs often cross species barriers to infect humans and have emerged to cause significant morbidity and mortality in the general population. The most recent examples are SARS-CoV, emerging in China in 2002 with 8000 infections and 800 deaths [16,17,18], and MERS-CoV, emerging in the Arabian Peninsula in 2012 [15,19,20]. Regarding SARS-CoV-2 evolution, Bat-CoV RaTG13, a bat CoV, is the closest known virus to SAR-CoV-2 and is predicted to have diverged 40–70 years ago [21]. The Pangolin-CoV shares 91.02% genome homology with SARS-CoV-2 and 90.55% genome homology with Bat-CoV RaTG13 and is the second closest relative to SARS-CoV-2 [22]. However, the exact identification of the zoonotic intermediate host for the evolutionary development of SARS-CoV-2 is unknown and will require further investigation.

Current analysis shows that diabetes, hypertension, cardiovascular diseases (CVDs), smoking, and chronic obstructive pulmonary disease (COPD) are among the most prevalent underlying diseases in patients hospitalized with COVID-19. Populations at higher risk for COVID-19 are more likely to become ill, need critical care, including mechanical ventilation, and die of the disease [23]. The general risk factors for COVID-19 include failure to comply with social distancing measures, restrictive population movements, and governmental and infectious disease guidelines for community protections to mitigate or prevent viral transmission [24]. It has been estimated that 45.4% of US adults are at increased risk for complications from COVID-19 due to CVD, diabetes, respiratory disease, hypertension, or cancer; this rate increases with age and may vary from state to state [25]. A report by Chen et al. describes the clinical course of the first 799 critically ill COVID-19 patients admitted to an isolation ward in Wuhan, China [26]. The authors compared the clinical profiles of 113 patients (14.4%) who have died of COVID-19 with those of 161 patients who recovered, finding that those who died were on average 17 years older (with no deaths among those under age 40 and 16.8% of deaths among those 40–60 years of age) [26]. Most of the patients who died were male and more likely to have a comorbidity, such as diabetes, hypertension, CVD, or chronic lung disease [26].

In the US, the disparity in COVID-19 mortality among minority populations is well known. It has become increasingly evident that the race or socioeconomic status of individuals affects their access to quality healthcare in the US, which may be the defining factor in acquiring COVID-19 disease, and may determine whether they survive. In the early stages of the pandemic, unsubstantiated rumors suggested that African Americans (AAs) were somehow immune to the adverse effects of COVID-19. Later, as transmission of the virus increased, especially in densely populated areas of the country, it became clear that COVID-19 disproportionately impacted AAs and Hispanics/Latinos (H/Ls) when compared with non-Hispanic Whites (NHWs) from the same communities. In addition, it was observed that mortality associated with COVID-19 was higher among AAs and H/Ls when compared with NHWs. In this review, the author examines the clinical risk factors for COVID-19 acquisition and adverse diseases that are disproportionate among minority populations and the disproportionate impact of COVID-19 on minority communities in the US. In addition, the author discusses interventions to mitigate clinical factors contributing to COVID-19 morbidity and mortality among minority populations in the US and briefly discusses policy changes that could combat health inequities during the COVID-19 crisis and beyond.

## 2. Disproportionate Impact of Coronavirus Disease 2019 (COVID-19) on Minority Communities in the US

The health inequities in the US that impact minority communities were well in place prior to the COVID-19 pandemic. These inequities have become more evident in some cities and states. In Chicago, AAs make up 30% of the population; yet, they represent 50% of COVID-19 cases and approximately 70% of COVID-19 deaths, most of which are concentrated in small numbers of the most vulnerable communities [27]. In the states of Louisiana and Michigan, AAs are impacted disproportionately by COVID-19 deaths [28]: Blacks represent 32.2% and 14%, respectively, and account for 70.5% and 40% of COVID-19 deaths, respectively [29]. In New York City, which had the largest number of COVID-19 cases and the highest number of deaths due to COVID-19 in the US, minority communities disproportionately are impacted, as AAs and H/Ls account for 22% and 29% of the population, respectively, and account for 28% and 34% of deaths, respectively [30]. An examination of 131 predominantly AA counties shows a COVID-19 infection rate of 137.5 per 100,000 and a death rate of 6.3 per 100,000, which is three times higher than the predominant NHW counties. Moreover, the death rate for the AA counties were found to be six times higher than the rate observed in predominant white counties. Taken together, a disproportionate burden of COVID-19 morbidity and mortality, which will require further investigation, exists among minority communities in the US. It is evident that social determinants of health play a critical role in population-level health disparities beyond the comorbidities associated with COVID-19 in minority communities.

In the early stages of the pandemic in the US, dismissing COVID-19 as not infecting AAs may have created a lack of awareness and best practices, including proper hand hygiene, use of masks in public places, and social distancing and physical isolation, likely contributing to SARS-CoV-2 transmission in these communities [31]. Even were that not the case, among vulnerable populations with low socioeconomic status, these transmission-mitigating practices are difficult to maintain over time.

## 3. Clinical Risk Factors for COVID-19 Acquisition and Adverse Disease That Are Disproportionate among Minority Populations in the US

### 3.1. Diabetes and COVID-19 Patients

Racial and ethnic disparities in the prevalence of type 2 diabetes (T2D) among adult minority populations have been documented [32,33]. More recently, the global epidemic of childhood obesity has contributed greatly to the higher prevalence of T2D among adolescents who have more progressive clinical presentations of chronic kidney disease (CKD), CVD, diabetes-related eye disease, and poor glycemic control over time [34]. Higher rates of T2D are seen among minority youth when compared with NHW youth [35]. Approximately 80% of youth with T2D are from minority backgrounds [36]. In addition, health disparities for T2D are present in adults and youth among racial and ethnic minorities, and this likely will impact their response to COVID-19.

**Figure 1:**
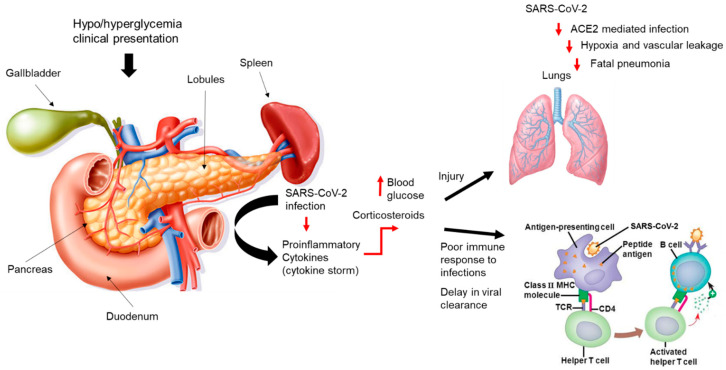
**Diabetes and increased risk for severe coronavirus disease 2019 (COVID-19).** Hypothetical model of uncontrolled glycemia in diabetic patients and increased risk for complications due to COVID-19. Patients who clinically present with normal or high blood pressure may be subject to undue complications related to severe acute respiratory syndrome coronavirus 2 (SARS-CoV-2) infection. This is an illustration of the pancreas, responsible for insulin production and regulation and the immediate surrounding tissue and organs. Once infected with SARS-CoV-2 some patients will experience increased inflammation in the form of a cytokine storm. Corticosteroids often are prescribed to suppress inflammation but also are known to induce high glucose levels in the blood of both hypoglycemic and hyperglycemic patients. High blood glucose levels have been implicated in pulmonary injury and may affect the immune response, resulting in poor or delayed viral clearance. The degree of lung injury will include Angiotensin-converting enzyme 2 (ACE2)-mediated infection by SARS-CoV-2 that leads to hypoxia, vascular leakage, and potentially fatal pneumonia. TCR (T-cell receptor), CD4 (cluster designation 4), MHC (Major Histocompatibility). Adapted from Fraussen J et al. [40].

COVID-19 patients with diabetes are at increased risk of having adverse clinical complications, including death [37,38]. Maintaining glycemic control in COVID-19 patients is essential, as hyperglycemia could affect pulmonary function, the immune response to infection, and the development of the pro-inflammatory cytokine storm associated with more severe clinical disease (Figure 1). The use of corticosteroid therapy to combat inflammation in COVID-19 patients also may increase glucose levels in 80% of patients with diabetes as well as patients without diabetes [39] (Figure 1).

### 3.2. Hypertension and COVID-19 Patients

**Figure 2:**
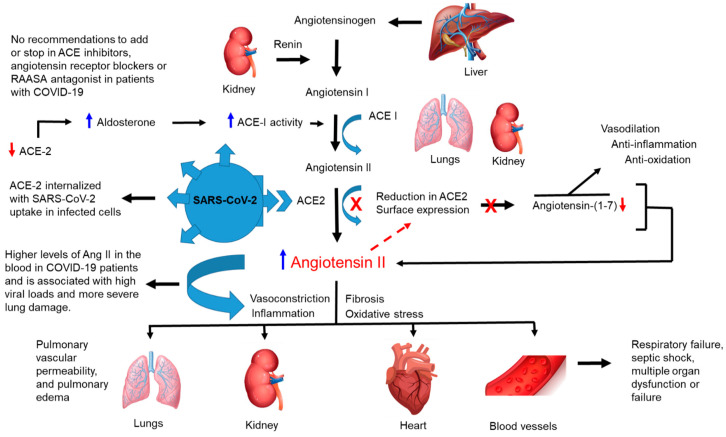
**Hypertension and increased risk for severe COVID-19 disease.** Hypothetical model of uncontrolled blood pressure in patients with hypertension and increased risk for complications due to COVID-19. The liver produces angiotensinogen, a peptide hormone that causes vasoconstriction that increases blood pressure. Angiotensinogen is converted to angiotensin I by renin from the kidneys then is converted to angiotensin II by the action of ACE (ACE-I). Normally angiotensin II (Ang. II) is converted to angiotensin-1–7 by the monocarboxypetidase ACEI homology ACE2. However, upon infection with SARS-CoV-2 the ACE2 protein serves as the entry receptor for the virus and is internalized in the endosome with SARS-CoV-2 during membrane fusion and uptake by infected cells. This leads to significant reduction in ACE2 surface expression and concomitant increase in angiotensin II, further leading to vasoconstriction that impacts blood pressure, inflammation, fibrosis, and oxidative stress in infected cells and tissues in multiple organs. Even more, the downregulation of ACE2 leads to the upregulation of aldosterone, a steroid hormone produced by the zona glomerulosa of the adrenal cortex, which increases the activity of ACE1. This action leads to higher levels of angiotensin II and an overall suppression of angiotensin-1–7, which is designed to mitigate the effects of angiotensin II via vasodilation, anti-inflammatory effects, and anti-oxidation. These levels of angiotensin II may result in MOD or failure. RAASA (renin-angiotensin-aldosterone system antagonist). ACE I (Angiotensin-converting enzyme 1).

The WHO concludes that hypertension is the most important risk factor for death and disability worldwide, affecting more than 1 billion people and causing an estimated 9.4 million deaths annually [41]. AA adults have the highest prevalence of hypertension in the US, affecting 40.8% of men and 41.5% of women, in contrast to significantly lower rates in NHW, non-Hispanic Asian (NHA), and H/L men and women [42]. Hypertension is the most significant factor that directly contributes to disparities in CVD and renal disease among AAs compared with NHWs [42]. In a study by Zhou et al., the most common comorbidities associated with adverse clinical outcomes in COVID-19 patients were hypertension (30%), diabetes (19%), and coronary heart disease (8%) [43]. In a separate study by Wu, the most common comorbidities associated with COVID-19 patients who developed acute respiratory distress syndrome were hypertension (27%), diabetes (19%), and CVD (6%) [44]. In these two major studies, hypertension was found to be the most significant comorbidity associated with the most severe complications from COVID-19. Hypertension is not known to be causative in COVID-19 pathobiology, and elderly patients are more likely to be hypertensive and are known to be at greater risk of severe disease. It also remains unclear whether medications used to treat hypertension, such Angiotensin-converting enzyme inhibitors (ACEIs), have a role in acquisition or progressive development of COVID-19 in patients. In humans, the liver produces angiotensinogen, which is converted to angiotensin I by renin from the kidneys, and angiotensin I is converted to angiotensin II by the action of ACE (Figure 2). Normally, angiotensin II is converted to angiotensin-1–7 by the monocarboxypetidase ACE2 (Figure 2). However, upon infection with SARS-CoV-2, the ACE2 protein serves as an entry receptor for the virus and is internalized with SARS-CoV-2 during membrane fusion and uptake by infected cells (Figure 2). This leads to a significant reduction in ACE2 surface expression and a concomitant increase in angiotensin II, further leading to vasoconstriction that impacts blood pressure and causes inflammation, fibrosis, and oxidative stress in infected tissues within multiple organs (Figure 2). Thus, the downregulation of ACE2 leads to the upregulation of aldosterone, which increases the activity of ACE1, which again leads to higher levels of angiotensin II and an overall suppression of angiotensin-1–7, designed to mitigate the effects of angiotensin II via vasodilation, anti-inflammatory effects, and anti-oxidation (Figure 2). Taken together, this may result in multi-organ dysfunction (MOD) or failure. However, it is known that SARS-CoV-2 binds to the ACE2 receptor, mainly in the lung, to enter cells [45,46]. Therefore, it remains controversial whether treatment of hypertension with ACEIs is beneficial or counter-productive in COVID-19 patients. Studies have shown the ACEIs have a protective anti-inflammatory effect in the lung, and soluble ACEIs could bind free virus and serve as therapeutics to reduce virus load [47,48,49]. If corticosteroid therapy is recommended for COVID-19 patients, glucose levels should be monitored carefully to avoid pulmonary and immune dysfunction [50,51] (Figure 1). Controversy surrounds the discontinuation of angiotensin receptor blockers (ARBS) and ACEIs for diabetes and hypertension treatment in COVID-19 patients [52]. However, in a recent study involving Renin-angiotensin-aldosterone system inhibitors, it is recommended that these drugs be continued in patients evaluated for COVID-19 to avoid excess cardiovascular risk [53]. Optimal care of diabetes patients with COVID-19 should involve careful monitoring of corticosteroid therapy when warranted, monitoring of regular blood glucose, and avoiding inappropriate discontinuation of ARBS and ACEIs that may increase morbidity in COVID-19 patients. ACE2 also is expressed in heart, kidneys, vascular endothelium, and intestinal epithelium, supporting the notion that virus interaction with several organ systems could lead to MOD, which may be observed in COVID-19 patients [54,55].

### 3.3. Cardiovascular Disease and COVID-19 Patients

African Americans are two to three times more likely than NHWs to die of preventable CVD and stroke even when accounting for the general decline in CVD mortality [56]. From 2011–2014, a study found that the prevalence of hypertension among AA adults (41.2%) was higher than among NHW (28.0%), NHW (24.9%), and Hispanic (25.9%) adults [57]. Medication non-adherence is one of the greatest challenges to reducing health disparities associated with CVD morbidity and mortality [58]. Poor management of hypertension is linked to increased risk of CVD, stroke, and CKD; hypertension management among AAs has been shown to be lower when compared with NHWs [59].

**Figure 3:**
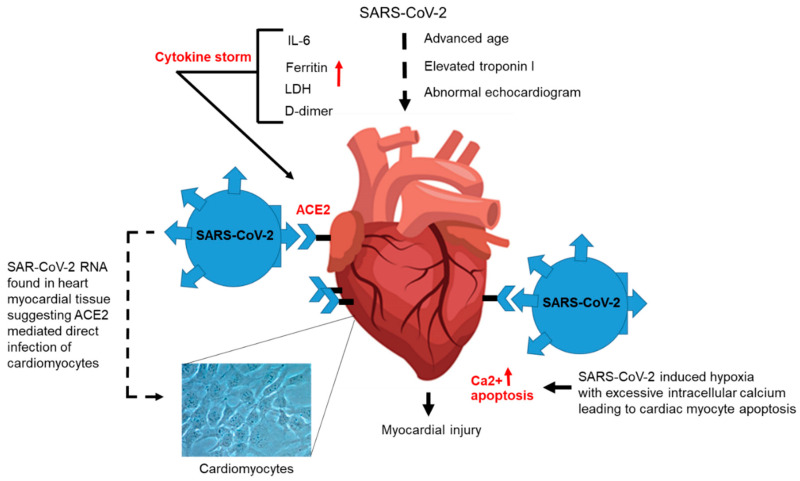
**Cardiovascular disease (CVD) and increased risk for severe COVID-19 disease.** Hypothetical model of patients with CVD and increased risk for complications due to COVID-19. CVD patients with advanced age and elevated troponin I levels, and who have abnormal echocardiograms, are at high risk for developing more severe COVID-19 disease. These patients have been shown to have increased blood levels of IL-6, ferritin, LDH, and D-dimer, which are associated with the cytokine storm that likely would contribute to cardiac injury. High levels of ACE2 are known to be expressed on cardiomyocytes that could result in direct infection of heart tissue by SARS-CoV-2, which is thought to induce hypoxia leading to increase calcium levels resulting in apoptosis and death of cardiomyocytes; this likely would contribute to myocardial injury.

CVD is a common comorbidity in patients with COVID-19 and is associated with patients who have the most severe disease [60]. A recent meta-analysis of eight studies from China that included 46,248 COVID-19-infected patients showed the most prevalent comorbidities were hypertension (17 ± 7%, 95% confidence interval (CI) 14–22%) and diabetes (8 ± 6%, 95% CI 6–11%), followed by CVDs (5 ± 4%, 95% CI 4–7%) [61]. COVID-19 interacts with the cardiovascular system, inducing myocardial injury. Patients with advanced age and elevated troponin I levels have abnormal echocardiograms and are at the greatest risk for developing more severe COVID-19 disease (Figure 3). These patients have been shown to have increased blood levels of interleukin-6 (IL-6), ferritin, Lactic Acid Dehydrogenase (LDH), and fibrin degradation product (D-dimer), which are associated with the cytokine storm that likely would contribute to cardiac injury (Figure 3). For patients with cardiac insufficiency who have underlying heart disease, SARS- CoV-2 infection may lead to adverse clinical disease and death. Aging, ACE2 levels, waning of the immune response, and host factors that become pronounced in patients with CVD have been considered as possible explanations for the severe disease course observed in COVID-19 patients. In a study in Wuhan, China, which included 138 COVID-19 patients, researchers observed laboratory evidence of cardiac injury indicated by elevated troponin I levels, as well as abnormal echocardiograms in 7.2% of patients (10) overall, and 22% (30) who required intensive care unit (ICU) attention [62]. Zheng et al., reported that 12% of COVID-19 patients who died without known CVD risk clinically presented with elevated levels of troponin I or cardiac arrest during their hospitalization [63]. The connection between COVID-19 and CVD remains unclear; however, it has been suggested that direct infection of the heart occurs via ACE2 expression on myocardial tissue, with supporting evidence in a murine model demonstrating ACE2-dependent myocardial infection of SARS-CoV-2 [64] (Figure 3). In addition, SARS-CoV-2 RNA was detected in heart tissue from 35% of patients who died during the SARS-CoV-2 outbreak in Toronto [65]. A cytokine storm and calcium-dependent apoptosis of cardiomyocytes are among other mechanism that could link CVD and COVID-19 [66] (Figure 3). ACE2-related signaling pathways also may have a role in heart injury.

Heart transplant patients are expected to be especially vulnerable to SARS-CoV-2 infection due to immunosuppression; however, in a small study performed with 87 heart transplant recipients in Wuhan, China, no evidence of a higher risk of infection with SARS-CoV-2 was found when precautionary measures were taken [67]. Recommended guidelines reported as Guidance for Cardiothoracic Transplant and Mechanical Circulatory Support Centers regarding SARS CoV-2 infection and COVID-19 have been established and must be followed when performing heart transplantation [68]. It is recommended that patients continue heart transplantation without changes in immunosuppression, provided the recipient has not tested positive for SARS-CoV-2 and has not had exposure to or symptoms of COVID-19 in the prior two to four weeks [69] It is also recommended to avoid donors with known or suspected COVID-19, and if donors had COVID-19, they should be COVID-19-free (as indicated by polymerase chain reaction) for at least 14 days, owing to the incubation period of ~5 days and onset of symptoms in ~11.5 days [70].

### 3.4. Pulmonary Disease and COVID-19

The 2013 American Thoracic Society/European Respiratory Society Committee, and similarly Healthy People 2020, published a policy statement defining disparities in respiratory health as closely linked to racial ancestry, social, economic, and/or environmental differences [71]. Health disparities in respiratory diseases are 14 times higher in minority populations with the lowest socioeconomic status when compared with populations with the highest socioeconomic status [71]. Globally, a disproportionate burden of Chronic obstructive pulmonary disease (COPD) is present among minority communities due to low socioeconomic status, differences in health behaviors, occupational and social environmental exposures, prenatal and childhood exposures, respiratory tract infections, and tobacco use. These factors are associated with the risk of developing COPD and are associated with poor clinical outcomes related to COPD clinical presentations [72]. According to the WHO, >90% of COPD deaths occur in low-income and middle-income countries [73]. Social determinants of health, including economic stability, education, access to health care, and environment, play a critical role in establishing health equity that will reduce disparity-related respiratory diseases, such as COPD, during a lifespan [74,75].

**Figure 4:**
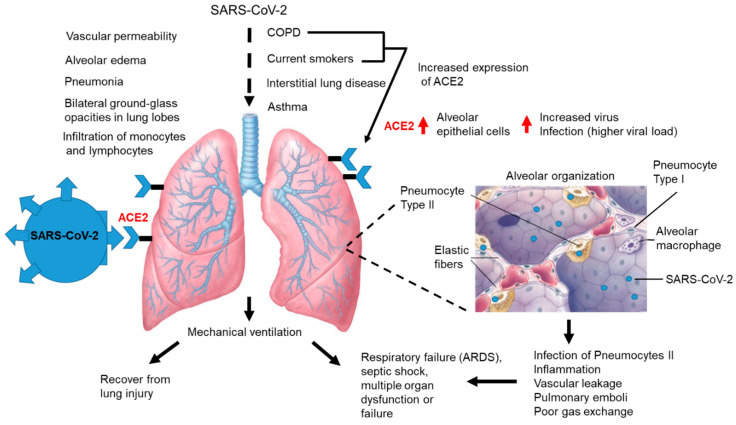
**Lung disease and increased risk for severe COVID-19 disease.** Hypothetical model of patients with pulmonary disease and increased risk for complications due to COVID-19. SARS-CoV-2 infection via ACE2 entry into alveolar epithelial cells in patients with COPD who smoke may lead to increased surface-expression ACE2 on lung epithelium that may increase the rate of infection in the lung and contribute to higher viral loads. Shown is the alveolar organization and resident cells that include pneumocytes Type I, pneumocytes Type II and alveolar macrophages. Elastic fibers are also shown and SARS-CoV-2 particles are shown as blue ovals. Alveolar cells pneumocytes type II are highly permissive for SARS-CoV-2 infection resulting in alveolar dysfunction, inflammation, vascular leakage, development of pulmonary emboli, and acute respiratory distress from poor gas exchange. Progressive viral infection and potential cytokine storm leads to pulmonary vascular leakage, alveolar edema, and monocyte infiltration at sites of infection, resulting in reduced lung function. Patients often require mechanical ventilation increasing the risk of delayed recovery, respiratory failure, septic shock, and MOD or failure. ARDS (Acute respiratory distress syndrome).

COPD is a condition that predisposes COVID-19 patients to worse clinical outcomes [76]. Smokers and individuals with COPD have increased airway expression of the ACE2 receptor for SAR-CoV-2 [77]. COVID-19 patients with COPD who are current smokers and have other types of lung disease, including asthma, have poor clinical outcomes (Figure 4). SARS-CoV-2 infection via ACE2 entry into the alveolar epithelial cells of COPD patients who smoke may lead to increased surface-expression ACE2 on lung epithelium, which may increase the rate of infection in the lung and contribute to higher viral loads (Figure 4). Alveolar cells pneumocytes type II are highly permissive for SARS-CoV-2 infection resulting in alveolar dysfunction, inflammation, vascular leakage, development of pulmonary emboli, and acute respiratory distress from poor gas exchange (Figure 4). Progressive viral infection and the pro-inflammatory conditions lead to pulmonary vascular leakage, alveolar edema, monocyte infiltration, and pneumonia (Figure 4). Patients often require mechanical ventilation resulting in delayed recovery, respiratory failure, septic shock, or MOD or failure (Figure 4).

Differential expression of ACE2 may help to explain the discrepancy in viral pathology associated with COVID-19. The production of ACE2 among minority populations who smoke or have COPD may partially explain the differences in COVID-19 rates of morbidity and mortality among AAs compared with NHWs. Leung et al. examined gene expression levels of ACE2 in the airways of individuals with and without COPD and found that active cigarette smoking and COPD upregulate ACE2 expression in lower airways and could contribute to the difference in disease burden observed among COVID-19 patients [77]. These findings were supported in a rat model that showed smoke exposure resulted in increased expression and activity of ACE2 in the airways [78]. Individuals with uncontrolled asthma also appear to be at increased risk of a more severe course of COVID-19 infection [79]. Lung function tests and the use of nebulizers as part of COPD and asthma management should be performed with caution due to the risk of virus aerosolization and potential transmission during these procedures [79].

## 4. Interventions to Mitigate Clinical Factors Contributing to COVID-19 Morbidity and Mortality among Minority Populations in the US

### 4.1. Diabetes and COVID-19 Mitigation Strategies

Disparities in T2D among minority adults have been pervasive in diabetes complications, glycemic control, and diabetes care [80]. Glycemic control in all patients with diabetes is critical for avoiding complications such as CKD, CVD, and diabetes-related eye disease. It is projected that racial and ethnic disparities in T2D prevalence will persist until 2050 [81]. One of the most significant challenges in mitigating the effects of diabetes on the health and wellness of minority populations in the US is improving basic social determinants of health, including low socioeconomic status, poor access to educational opportunities, conditions of poverty, excess life stressors, poor health knowledge, and limited access to quality and affordable health care. Strategies to mitigate diabetes among minority populations at greater risk for severe COVID-19 disease would involve improving glycemic control among AAs and H/Ls, which is a major problem [82]. Management of hypertension has been problematic among minority populations with diabetes. It has been reported that 71% of adults with diabetes have high blood pressure, and blood pressure control among diabetics is essential to reduce the risk of developing retinopathy and neuropathy associated with diabetes [83]. AAs are more likely to have significantly higher, uncontrolled blood pressure than NHWs [83]. Obesity and a sedentary lifestyle should be avoided among diabetics [83]. The increased burden of diabetes among racial and ethnic minorities. Diabetes is higher among Black or African American, H/Ls, and American Indian individuals as compared to NHW [84]. AAs experiencing household or neighborhood-level poverty are at a higher risk of developing diabetes [84]. The rates of diabetes in the Southern U.S. is higher than the national average [85].

This will require overall improvements in social determinants of health that tend to predispose these populations to the worst diabetes outcomes often superimposed on the pathogenesis of COVID-19 disease burden.

### 4.2. Hypertension and COVID-19 Mitigation Strategies

Among racial and ethnic minorities, hypertension prevalence in the US is highest among AAs, who have been shown to have less control of the disease when compared with NHWs [86,87,88,89]. Pharmacotherapy in the form of thiazide-type diuretics, calcium channel blockers, ACEIs or Angiotensin II receptor blockers (ARBs), and lifestyle changes are essential for hypertension control [61]. Medication adherence plays a critical role in the long-term management of hypertension, especially in minority communities, and is directly related to racial and ethnic disparities in CVD, stroke, and chronic kidney disease (CKD). Ferdinand et al. report that strategies such as direct patient engagement, consumer-directed health care, utilization of patient portals, smart apps and text messages, digital pillboxes, pharmacist-led engagement, and cognitive-based behavior, could be important for improving medical adherence and reducing disparities in hypertension and its related complications [90]. The Centers for Disease Control and Prevention (CDC) reports that 75% of individuals who died of COVID-19 disease were older than 65 years of age. Age-related increase in systolic blood pressure suggests that 90% of adults in the US will develop hypertension in their lifetime. This age-related increase in blood pressure likely contributes to increased age-related mortality among the elderly with COVID-19.

### 4.3. Cardiovascular Disease and COVID-19 Mitigation Strategies

It has been proposed that CVD is more prevalent in older patients, as these patients are more likely to have impaired immune systems that loses the ability to responds effectively to infections and the reduced levels of ACE2 observed among the elderly may predispose older patients to more severe complications of COVID-19 due to loss of the protective effects of ACE2 to regulate and control inflammation.

Therefore, patients with CVD should be subjected first to preventive measures, such as monitoring for elevated cardiac biomarkers, and if necessary, should be isolated from other patient populations that would place them at higher risk. In addition, a report from the National Health Commission of China revealed that almost 12% of COVID-19 patients without known CVD risk had elevated troponin levels or cardiac arrest during hospitalization [91]. This would suggest that SARS-CoV-2-mediated infection may lead directly to myocardial dysfunction. Potential benefit or harm from ACEIs and ARBs, commonly prescribed for cardiovascular disorders, is still under investigation due to lack of available evidence.

### 4.4. Chronic Obstructive Pulmonary Disease and COVID-19 Mitigation Strategies for Underserved Populations

Individuals with COPD and confirmed COVID-19 are at greater risk of severe complications and death than individuals without COPD. Optimal testing and diagnostics, as well as management of individuals with COPD in underserved communities, should be made available for early intervention and treatment. These individuals should be screened for other comorbidities that are associated with the development of COPD, such as asthma, lung infections, and other related respiratory diseases. Affordability and access to treatments and palliative care should be made available to low-income COPD patients. Furthermore, COPD patients should be allowed access to optimal life-extending treatments for their disease, including medical specialists, more effective drug treatments, smoking cessation counseling, supplemental oxygen, and non-invasive ventilation that often are unaffordable for these vulnerable populations [92]. Having COPD and being a current smoker may greatly increase the risk of complications mortality from COVID-19. Interventions in COPD patients who smoke should include smoking cessation, as it has been demonstrated to improve pulmonary function in younger smokers compared with older smokers who have sustained cumulative lung damage over time [92]. Essential workers, who often are from minority communities and face occupational hazards, are at greater risk for developing occupation-related COPD.

Understanding clinical risk factors and their mechanisms can benefit disparity populations by identifying at risk individuals early and developing early intervention strategies that are designed to manage these risk factors in the context of clinical trials where access to care and drug compliance can be controlled. The benefits of understanding these mechanisms could also provide information that contributes to innovative therapies that identify selective targets within these pathways among disparity populations based on unique genetic profiles. Finally, an understanding of these mechanisms could lead to changes among health providers to be more aggressive in their care for these patients as clinical trial data become available, leading changes in health policy for underserved populations.

## 5. Policy Changes to Combat Health Inequities during COVID-19 Crisis and Beyond

Changes in public policy are essential to combat the long-standing problems associated with health inequities in our health care system. These inequalities are more pronounced during a health care crisis, such as the current COVID-19 pandemic. Addressing these inequities would require a government-appointed race/inequity task force that is designed to implement pre-determined standards of care in minority communities at an early stage in a medical crisis. In addition, special provisions should be made for essential workers, many of whom are underserved, to be adequately equipped and compensated for vital services performed to maintain public health standards. Adequate funding should be established to support these initiatives from both the public and private sectors to avoid disruptions in the readiness of our workforce, supply chains, and health care system to prevent unforeseen economic crisis. New policies must be flexible enough to accommodate changes in our scientific understanding of these emerging pathogens and the development of efficacious interventions to protect the public, including our most vulnerable populations. These policies would require a bipartisan commitment from government officials, ending a “wait and see if it goes away” strategy by replacing previous tactics with a standard plan of action, which would save lives and reduce the overall burden on our economy. Policy changes would include the elimination of inequitable treatment within our health care system.

## 6. Practices That May Enhance the Effectiveness of Clinicians When They Engage Disparity Populations

Doctor–patient relationships that include individuals from underserved communities in the US can be difficult due to systemic racism and implicit racial bias that has contributed to historic distrust in minority communities for health care providers. Physicians could engage minority patients in ways that will help to assure them that they will receive the best possible care. For those minority populations who are essential workers that are at higher risk for Covid-19 because of pre-existing health conditions, physicians should aggressively make them aware of the risks as well as precautionary measures they must take to avoid infection. Aggressive strategies to help minority patients at higher risk understand the seriousness of COVID-19 disease could include direct mailings, providing patients with samples of masks and sanitizers, COVID-19 office placards, COVID-19 infomercials in waiting areas, wellness checks, and COVID-19 information for family members and caretakers. The Social Determinants of Health (SDOH) for these individuals are directly linked to the development of risk factors that predispose them to more severe COVID-19 disease. Healthcare providers and patient navigators that have relationships with community based organizations (CBOs) that work to improve SDOH among underserved communities could be an important option to support the long term health and wellness of disparity populations. Front line health care providers in the Covid-19 pandemic are being pushed to the limits of human endurance and are often overwhelmed which can trigger disparate responses to minority communities. Policies need to be in place that confer oversight for when this occurs. Physicians should be surveyed in ways to recognize implicit racial bias when caring for minority patients and be encouraged to take measures to constructively modify their behavior. In addition, there is a great need for more nurses and other medical practitioners to support physicians during this pandemic. Some of these challenges can be met with volunteers that have past medical training as well as military medical staff. Essential workers, many of whom are from minority communities are often forced to experience increased risk of virus transmission because of economic hardships and the lack of personal protective equipment (PPE). The lack of PPE is directly linked to the likelihood of virus transmission to essential workers and, therefore, should be recognized as a necessity that should be prioritized by the federal government as an emergency declaration that would be fully funded throughout this pandemic and policies put in place in this declaration for timely ramping-up of PPE supplies and development of government-owned PPE stockpiles for a future medical crisis. A commission of physicians, and nurses should be established to advocate the need for PPE on behalf of essential workers at risk for COVID-19 and given the opportunity the make their case to policy makers.

Healthcare access is a core component of the SDOH for underserved communities and poor access to affordable healthcare is a major driver of health disparities in the US. These disparity populations are heavily impacted by this pandemic. The Affordable Care Act (ACA) represents a lifeline for the working poor and their families without health insurance. The ACA should be maintained and arguably updated or revised to meet the changing needs of participants. The ACA is a critical component of our existing healthcare infrastructure that directly addresses health inequities in the US and is not perfect in content and yet should be preserved. The existing political climate for legislation to directly address longstanding US racial/ethnic inequities in healthcare as well as other SDOH such as education, housing, and a living wage will require a continuance of the grass roots call for change and the acceptance by policy makers that these changes need to become law in America.

## 7. Conclusions

Longstanding health disparities such as diabetes, hypertension, CVD, and pulmonary disease among minority populations in the US may serve to predispose these communities to SARS-CoV-2 infection and increased risk for clinically severe COVID-19. The underlying social determinants of health and standards of care in minority communities must be improved to end these disparities. Improvements will require changes in governmental policy and a long-term commitment to minority communities that includes early interventions and prevention strategies to reduce or eliminate major healthy disparities on the way to achieving health equity.

## References

[1] Bassetti M., Vena A., Giacobbe D.R. (2020). The novel Chinese coronavirus (2019-nCoV) infections: Challenges for fighting the storm. Eur. J. Clin. Investig..

[2] Lai C.-C., Shih T.-P., Ko W.-C., Tang H.-J., Hsueh P.-R. (2020). Severe acute respiratory syndrome coronavirus 2 (SARS-CoV-2) and coronavirus disease-2019 (COVID-19): The epidemic and the challenges. Int. J. Antimicrob. Agents.

[3] Li Q., Guan X., Wu P., Wang X., Zhou L., Tong Y., Ren R., Leung K.S.M., Lau E.H.Y., Wong J.Y. (2020). Faculty Opinions recommendation of Early Transmission Dynamics in Wuhan, China, of Novel Coronavirus-Infected Pneumonia. Faculty Opinions—Post-Publication Peer Review of the Biomedical Literature. N. Engl. J. Med..

[4] Huang C., Wang Y., Li X., Ren L., Zhao J., Hu Y. (2020). Clinical features of patients infected with 2019 novel coronavirus in Wuhan, China. Lancet.

[5] Woo P.C., Lau S.K.P., Lam C.S.F., Lau C.C.Y., Tsang A.K.L., Lau J.H.N., Bai R., Teng J.L.L., Tsang C.C.C., Wang M. (2012). Discovery of Seven Novel Mammalian and Avian Coronaviruses in the Genus Deltacoronavirus Supports Bat Coronaviruses as the Gene Source of Alphacoronavirus and Betacoronavirus and Avian Coronaviruses as the Gene Source of Gammacoronavirus and Deltacoronavirus. J. Virol..

[6] Chairez R., Hollinger B., Melnick J.L., Dreesman G.R., Ghendon Y., Porubel L., Sasaki Y., Sasaki R., Cohen G.H., Pizer L.I. International Committee on Taxonomy of Viruses. Virus Taxonomy: 2017 Release. https://talk.ictvonline.org/taxonomy/.

[7] Du Toit A. (2020). Outbreak of a novel coronavirus. Nat. Rev. Genet..

[8] Rothan H.A., Byrareddy S. (2020). The epidemiology and pathogenesis of coronavirus disease (COVID-19) outbreak. J. Autoimmun..

[9] Chan J., To K.K.W., Tse H., Jin D.-Y., Yuen K.-Y. (2013). Interspecies transmission and emergence of novel viruses: Lessons from bats and birds. Trends Microbiol..

[10] Hui D.S., Azhar E.E., Madani T.A., Ntoumi F., Kock R., Dar O., Ippolito G., McHugh T.D., Memish Z.A., Drosten C. (2020). The continuing 2019-nCoV epidemic threat of novel coronaviruses to global health—The latest 2019 novel coronavirus outbreak in Wuhan, China. Int. J. Infect. Dis..

[11] Fehr A.R., Perlman S. (2015). Coronaviruses: An Overview of Their Replication and Pathogenesis. Methods Mol. Biol..

[12] Weiss S.R., Navas-Martin S. (2005). Coronavirus Pathogenesis and the Emerging Pathogen Severe Acute Respiratory Syndrome Coronavirus. Microbiol. Mol. Boil. Rev..

[13] Letko M.C., Marzi A., Munster V.J. (2020). Functional assessment of cell entry and receptor usage for SARS-CoV-2 and other lineage B betacoronaviruses. Nat. Microbiol..

[14] Woo P.C.Y., Lau S.K.P., Chu C., Chan K.-H., Tsoi H.-W., Huang Y., Wong B.H.L., Poon R.W.S., Cai J.J., Luk W.-K. (2005). Characterization and Complete Genome Sequence of a Novel Coronavirus, Coronavirus HKU1, from Patients with Pneumonia. J. Virol..

[15] Chan J., Lau S.K.P., To K.K.-W., Cheng V.C.C., Woo P.C., Yuen K.-Y. (2015). Middle East Respiratory Syndrome Coronavirus: Another Zoonotic Betacoronavirus Causing SARS-Like Disease. Clin. Microbiol. Rev..

[16] Cheng V.C.C., Lau S.K.P., Woo P.C.Y., Yuen K.-Y. (2007). Severe Acute Respiratory Syndrome Coronavirus as an Agent of Emerging and Reemerging Infection. Clin. Microbiol. Rev..

[17] Zhong N.S., Zheng B.J., Li Y.M., Poon L.L.M., Xie Z.H., Chan K.H., Li P.H., Tan S.Y., Chang Q., Xie Liu Q.X. (2003). Epidemiology and cause of severe acute respiratory syndrome (SARS) in Guangdong, People’s Republic of China, in February 2003. Lancet.

[18] Kuiken T., Fouchier R.A.M., Schutten M., Rimmelzwaan G.F., Van Amerongen G., Van Riel D., Laman J.D., De Jong T., Van Doornum G., Lim W. (2003). Newly discovered coronavirus as the primary cause of severe acute respiratory syndrome. Lancet.

[19] Zaki A.M., Van Boheemen S., Bestebroer T., Osterhaus A., Fouchier R. (2012). Isolation of a Novel Coronavirus from a Man with Pneumonia in Saudi Arabia. N. Engl. J. Med..

[20] Van Boheemen S., De Graaf M., Lauber C., Bestebroer T.M., Raj V.S., Zaki A.M., Osterhaus A.D., Haagmans B.L., Gorbalenya A.E., Snijder E.J. (2012). Genomic Characterization of a Newly Discovered Coronavirus Associated with Acute Respiratory Distress Syndrome in Humans. mBio.

[21] Bezerra R.D.S., Valença I.N., Ruy P.D.C., Ximenez J.P.B., da Silva Júnior W.A., Covas D.T., Kashima S., Slavov S.N., Covas D.T. (2020). The novel coronavirus SARS-CoV-2: From a zoonotic infection to coronavirus disease 2019. J. Med. Virol..

[22] Zhang T., Wu Q., Zhang Z. (2020). Probable Pangolin Origin of SARS-CoV-2 Associated with the COVID-19 Outbreak. Curr. Biol..

[23] EJordan R., Adab P., Cheng K.K. (2020). Covid-19: Risk factors for severe disease and death. BMJ.

[24] Public Health England (2020). Guidance on Social Distancing for Everyone in the UK. https://www.gov.uk/government/publications/covid-19-guidance-on-social-distancing-and-forvulnerable-people/guidance-on-social-distancing-for-everyone-in-the-uk-and-protectingolder-people-and-vulnerable-adults.

[25] Adams M.L., Katz D.L., Grandpre J. (2020). Population-based estimates of chronic conditions affecting risk for complications from coronavirus disease, United States. Emerg. Infect. Dis..

[26] Chen T., Wu D., Chen H., Yan W., Yang D., Chen G., Ma K., Xu D., Yu H., Wang H. (2020). Clinical characteristics of 113 deceased patients with coronavirus disease 2019: Retrospective study. BMJ.

[27] Reyes C., Husain N., Gutowski C., St Clair S., Pratt G. Chicago’s Coronavirus Disparity: Black Chicagoans are Dying at Nearly Six Times the Rate of White Residents, Data Show. Chicago Tribune. https://www.chicagotribune.com/coronavirus/ct-coronavirus-chicago-coronavirus-deaths-demographics-lightfoot-20200406-77nlylhiavgjzb2wa4ckivh7mu-story.html.

[28] Thebault R., Ba Tran A., Williams V. The Coronavirus is Infecting and Killing Black Americans at an Alarmingly High Rate. Washington Post. https://www.washingtonpost.com/nation/2020/04/07/coronavirus-is-infecting-killingblack-americans-an-alarmingly-high-rate-postanalysis-shows/.

[29] Deslatte M. Louisiana Data: Virus Hits Blacks, People with Hypertension. US NewsWorld Report. https://www.usnews.com/news/best-states/louisiana/articles/2020-04-07/louisiana-data-virushits-blacks-people-with-hypertension.

[30] New York State Department of Health COVID-19 Fatalities. https://covid19tracker.health.ny.gov/views/NYS-COVID19-Tracker/NYSDOHCOVID-19Tracker-Fatalities?%3Aembed=yes&%3Atoolbar=no&%3Atabs=n.

[31] Pan A., Liu L., Wang C., Guo H., Hao X., Wang Q., Huang J., He N., Yu H., Lin X. (2020). Association of Public Health Interventions With the Epidemiology of the COVID-19 Outbreak in Wuhan, China. JAMA.

[32] McBean A.M., Li S., Gilbertson D.T., Collins A.J. (2004). Differences in diabetes prevalence, incidence, and mortality among the elderly of four racial/ethnic groups: Whites, blacks, hispanics, and asians. Diabetes Care.

[33] Mokdad A.H., Bowman B.A., Ford E.S., Vinicor F., Marks J.S., Koplan J.P. (2001). The continuing epidemics of obesity and diabetes in the United States. JAMA.

[34] Butler A.M. (2017). Social Determinants of Health and Racial/Ethnic Disparities in Type 2 Diabetes in Youth. Curr. Diab. Rep..

[35] Pinhas-Hamiel O., Zeitler P.S. (2007). Acute and chronic complications of type 2 diabetes mellitus in children and adolescents. Lancet.

[36] Klingensmith G.J., Connor C.G., Ruedy K.J., Beck R.W., Kollman C., Haro H., Wood J.R., Lee J.M., Willi S.M., Cengiz E. (2015). Presentation of youth with type 2 diabetes in the Pediatric Diabetes Consortium. Pediatr. Diabetes.

[37] Klonoff D.C., Umpierrez G.E. (2020). Letter to the Editor: COVID-19 in patients with diabetes: Risk factors that increase morbidity. Metabolism.

[38] Hill M.A., Mantzoros C., Sowers J.R. (2020). Commentary: COVID-19 in patients with diabetes. Metabolism.

[39] Yang J.-K., Feng Y., Yuan M.Y., Yuan S.Y., Fu H.J., Wu B.Y., Sun G.Z., Yang G.R., Zhang X., Wang L. (2006). Plasma glucose levels and diabetes are independent predictors for mortality and morbidity in patients with SARS. Diabetes Med..

[40] Fraussen J., Claes N., Van Wijmeersch B., Van Horssen J., Stinissen P., Hupperts R., Somers V. (2016). B cells of multiple sclerosis patients induce autoreactive proinflammatory T cell responses. Clin. Immunol..

[41] WHO (World Health Organization) (2013). A Global Brief on Hypertension. http://ish-world.com/downloads/pdf/global_brief_hypertension.pdf.

[42] Laurencin C.T., McClinton A. (2020). The COVID-19 Pandemic: A Call to Action to Identify and Address Racial and Ethnic Disparities. J. Racial Ethn. Health Disparities.

[43] Zhou F., Yu T., Du R., Fan G., Liu Y., Liu Z., Xiang J., Wang Y., Song B., Gu X. (2020). Clinical course and risk factors for mortality of adult inpatients with COVID-19 in Wuhan, China: A retrospective cohort study. Lancet.

[44] Wu C., Chen X., Cai Y., Xia J., Zhou X., Xu S., Huang H., Zhang L., Zhou X., Du C. (2020). Risk Factors Associated with Acute Respiratory Distress Syndrome and Death in Patients with Coronavirus Disease 2019 Pneumonia in Wuhan, China. JAMA Intern. Med..

[45] Wan Y., Shang J., Graham R., Baric R.S., Li F. (2020). Receptor Recognition by the Novel Coronavirus from Wuhan: An Analysis Based on Decade-Long Structural Studies of SARS Coronavirus. J. Virol..

[46] Hoffmann M., Kleine-Weber H., Schroeder S., Krüger N., Herrler T., Erichsen S., Schiergens T.S., Herrler G., Wu N.-H., Nitsche A. (2020). SARS-CoV-2 Cell Entry Depends on ACE2 and TMPRSS2 and Is Blocked by a Clinically Proven Protease Inhibitor. Cell.

[47] Imai Y., Kuba K., Rao S., Huan Y., Guo F., Guan B., Yang P., Sarao R., Wada T., Leong-Poi H. (2005). Angiotensin-converting enzyme 2 protects from severe acute lung failure. Nature.

[48] Batlle D., Wysocki J., Satchell K. (2020). Soluble angiotensin-converting enzyme 2: A potential approach for coronavirus infection therapy?. Clin. Sci..

[49] Schiffrin E.L., Flack J.M., Ito S., Muntner P., Webb R.C. (2020). Response to “COVID-19 and ACEI/ARB: Not Associated?”. Am. J. Hypertens..

[50] Singh A.K., Majumdar S., Singh R., Misra A. (2020). Role of corticosteroid in the management of COVID-19: A systemic review and a Clinician’s perspective. Diabetes Metab. Syndr. Clin. Res. Rev..

[51] Horby P., Lim W.S., Emberson J.R., Mafham M., Bell J.L., Linsell L., Staplin N., Brightling C., Ustianowski A., The RECOVERY Collaborative Group (2020). Dexamethasone in Hospitalized Patients with Covid-19—Preliminary Report. N. Engl. J. Med..

[52] Fang L., Karakiulakis G., Roth M. (2020). Are patients with hypertension and diabetes mellitus at increased risk for COVID-19 infection?. Lancet Respir. Med..

[53] Vaduganathan M., Vardeny O., Michel T., McMurray J.J., Pfeffer M.A., Solomon S.D. (2020). Renin–Angiotensin–Aldosterone System Inhibitors in Patients with Covid-19. N. Engl. J. Med..

[54] Tikellis C., Thomas M. (2012). Angiotensin-Converting Enzyme 2 (ACE2) Is a Key Modulator of the Renin Angiotensin System in Health and Disease. Int. J. Pept..

[55] Zhang H., Penninger J.M., Li Y., Zhong N., Slutsky A.S. (2020). Angiotensin-converting enzyme 2 (ACE2) as a SARS-CoV-2 receptor: Molecular mechanisms and potential therapeutic target. Intensive Care Med..

[56] Ferdinand K.C., Yadav K., Nasser S.A., Od H.D.C., Lewin J., Cryer D.R., Senatore F.F. (2017). Disparities in hypertension and cardiovascular disease in blacks: The critical role of medication adherence. J. Clin. Hypertens..

[57] Centers for Disease Control and Prevention/National Center for Health Statistics, National Health and Nutrition Examination Survey, 2011–2014. https://www.cdc.gov/nchs/data/databriefs/db220.pdf.

[58] Ritchey M.D., Chang A., Powers C., Loustalot F., Schieb L., Ketcham M., Durthaler J., Hong Y. (2016). Vital Signs: Disparities in Antihypertensive Medication Nonadherence Among Medicare Part D Beneficiaries—United States, 2014. MMWR. Morb. Mortal. Wkly. Rep..

[59] Holmes H.M., Luo R., Hanlon J.T., Elting L.S., Suarez-Almazor M., Goodwin J.S. (2012). Ethnic disparities in adherence to antihypertensive medications of medicare part D beneficiaries. J. Am. Geriatr. Soc..

[60] Clerkin K.J., Fried J.A., Raikhelkar J., Sayer G., Griffin J.M., Masoumi A., Jain S.S., Burkhoff D., Kumaraiah D., Rabbani L. (2020). Coronavirus Disease 2019 (COVID-19) and Cardiovascular Disease 2020. Circulation.

[61] Yang J., Zheng Y., Gou X., Pu K., Chen Z., Guo Q., Ji R., Wang H., Wang Y., Zhou Y. (2020). Prevalence of comorbidities and its effects in patients infected with SARS-CoV-2: A systematic review and meta-analysis. Int. J. Infect. Dis..

[62] Wang D., Hu B., Hu C., Zhu F., Liu X., Zhang J., Wang B., Xiang H., Cheng Z., Xiong Y. (2020). Clinical Characteristics of 138 Hospitalized Patients With 2019 Novel Coronavirus–Infected Pneumonia in Wuhan, China. JAMA.

[63] Zheng Y.-Y., Ma Y.-T., Zhang J.-Y., Xie X. (2020). COVID-19 and the cardiovascular system. Nat. Rev. Cardiol..

[64] Oudit G.Y., Kassiri Z., Jiang C., Liu P.P., Poutanen S.M., Penninger J., Butany J. (2009). SARS-coronavirus modulation of myocardial ACE2 expression and inflammation in patients with SARS. Eur. J. Clin. Investig..

[65] Booth C.M., Matukas L.M., Tomlinson G., Rachlis A.R., Rose D.B., Dwosh H.A., Walmsley S.L., Mazzulli T., Avendano M., Derkach P. (2003). Clinical Features and Short-term Outcomes of 144 Patients With SARS in the Greater Toronto Area. JAMA.

[66] Lang J.P., Wang X., Moura F.A., Siddiqi H.K., Morrow D.A., Bohula E.A. (2020). A current review of COVID-19 for the cardiovascular specialist. Am. Heart J..

[67] Ren Z.-L., Hu R., Wang Z.-W., Zhang M., Ruan Y.-L., Wu Z.-Y., Wu H.-B., Hu X.-P., Hu Z.-P., Ren W. (2020). Epidemiologic and clinical characteristics of heart transplant recipients during the 2019 coronavirus outbreak in Wuhan, China: A descriptive survey report. J. Hear. Lung Transplant..

[68] Guidance for Cardiothoracic Transplant and Mechanical Circulatory Support Centers regarding SARS CoV-2 infection and COVID-19: March 17, 2020. https://community.ishlt.org/HigherLogic/System/DownloadDocumentFile.ashx?DocumentFileKey=afb06f06-5d63-13d4-c107-d152a9f6cd46.

[69] American Society of Transplantation 2019-nCoV (Coronavirus): FAQs for Organ Transplantation. Updated Feb 29, 2020. https://www.myast.org/sites/default/files/COVID19%20FAQ%20Tx%20Centers%20030220-1.pdf.

[70] Lauer S.A., Grantz K.H., Bi Q., Jones F.K., Zheng Q., Meredith H.R., Azman A.S., Reich N.G., Lessler J. (2020). The Incubation Period of Coronavirus Disease 2019 (COVID-19) From Publicly Reported Confirmed Cases: Estimation and Application. Ann. Intern. Med..

[71] Schraufnagel D.E., Blasi F., Kraft M., Gaga M., Finn P.W., Rabe K.F. (2013). An Official American Thoracic Society/European Respiratory Society Policy Statement: Disparities in Respiratory Health. Am. J. Respir. Crit. Care Med..

[72] Pleasants R., Riley I.L., Mannino D.M. (2016). Defining and targeting health disparities in chronic obstructive pulmonary disease. Int. J. Chronic Obstr. Pulm. Dis..

[73] World Health Organization Chronic Obstructive Pulmonary Disease (COPD); Fact Sheet. March 2015. https://www.who.int/mediacentre/factsheets/fs315/en/.

[74] World Health Organization Commission on Social Determinants of Health—Final Report. Closing the Gap in a Generation: Health Equity Through Action on the Social Determinants of Health. http://www.who.int/social_determinants/thecommission/finalreport/en/.

[75] Zhao Q., Meng M., Kumar R., Wu Y., Huang J., Lian N., Deng Y., Lin S. (2020). The impact of COPD and smoking history on the severity of COVID-19: A systemic review and meta-analysis. J. Med. Virol..

[76] Alqahtani J.S., Oyelade T., Aldhahir A.M., Alghamdi S.M., Almehmadi M., Alqahtani A.S., Quaderi S., Mandal S., Hurst J.R. (2020). Prevalence, Severity and Mortality associated with COPD and Smoking in patients with COVID-19: A Rapid Systematic Review and Meta-Analysis. PLoS ONE.

[77] Leung J.M., Yang C.X., Tam A., Shaipanich T., Hackett T.-L., Singhera G.K., Dorscheid D.R., Sin D. (2020). ACE-2 expression in the small airway epithelia of smokers and COPD patients: Implications for COVID-19. Eur. Respir. J..

[78] Yilin Z., Yandong N., Faguang J. (2015). Role of angiotensin-converting enzyme (ACE) and ACE2 in a rat model of smoke inhalation induced acute respiratory distress syndrome. Burn.

[79] Daccord C., Touilloux B., Von Garnier C. (2020). [Asthma and COPD management during the COVID-19 pandemic]. Rev. Med. Suisse.

[80] Lanting L.C., Joung I.M., MacKenbach J.P., Lamberts S.W., Bootsma A.H. (2005). Ethnic Differences in Mortality, End-Stage Complications, and Quality of Care Among Diabetic Patients: A review. Diabetes Care.

[81] Copeland K.C., Zeitler P., Geffner M., Guandalini C., Higgins J., Hirst K., Kaufman F.R., Linder B., Marcovina S., McGuigan P. (2011). TODAY Study Group. Characteristics of adolescents and youth with recent-onset type 2 diabetes: The TODAY cohort at baseline. J. Clin. Endocrinol. Metab..

[82] G. American Diabetes Association (2015). Strategies for Improving Care. Sec. 1 In Standards of Medical Care in Diabetes-2015. Diabetes Care.

[83] Kurian A.K., Cardarelli K.M. (2007). Racial and ethnic differences in cardiovascular disease risk factors: A systematic review. Ethn. Dis..

[84] Barker L.E., Kirtland K.A., Gregg E.W., Geiss L.S., Thompson T.J. (2011). Geographic Distribution of Diagnosed Diabetes in the U.S. Am. J. Prev. Med..

[85] Gaskin D.J., Thorpe R.J., McGinty E.E., Bower K., Rohde C., Young J.H., LaVeist T.A., Dubay L. (2014). Disparities in Diabetes: The Nexus of Race, Poverty, and Place. Am. J. Public Health.

[86] Stephens J., Artiga S., Paradise J. (2014). Health Coverage and Care in the South in 2014 and Beyond.

[87] Ferdinand K.C., Nasser S.A. (2017). Management of Essential Hypertension. Cardiol. Clin..

[88] CDC (2020). Coronavirus Disease 2019 (COVID-19): People Who Are at Higher Risk for Severe Illness.

[89] Vasan R.S., Beiser A., Seshadri S., Larson M.G., Kannel W.B., D’Agostino R.B., Levy D. (2002). Residual Lifetime Risk for Developing Hypertension in Middle-aged Women and Men. JAMA.

[90] Jean S.S., Lee P., Hsueh P.R. (2020). Treatment Options for COVID-19: The Reality and Challenges Review. J. Microbiol. Immunol. Infect..

[91] Bimonte S., Crispo A., Amore A., Celentano E., Cuomo A., Cascella M. (2020). Potential Antiviral Drugs for SARS-Cov-2 Treatment: Preclinical Findings and Ongoing Clinical Research (Suppl.3). In Vivo.

[92] Maddocks M., Lovell N., Booth S., Man W.D.-C., Higginson I. (2017). Palliative care and management of troublesome symptoms for people with chronic obstructive pulmonary disease. Lancet.

